# Standardized vaccination practices for preterm infants: Egyptian experts’ consensus

**DOI:** 10.1186/s12879-025-12243-0

**Published:** 2025-12-19

**Authors:** Ahmed El Beleidy, Mohamed Ghandour, Adel Reyad, Amira Edris, Gamal Samy, Hesham Abdel-Hady, Hisham Awad, Khalil AbdelKhalek, Mostafa El-Saied, Mohamed Omar, Moataza Bashir, Mourad Alfy Tadros, Mostafa Mohammady, Noha Gebril, Ranya Aly Hegazy, Safaa Shafik Imam, Sherif Elanwary, Walaa Adel Mansour

**Affiliations:** 1 Pediatric Department, Faculty of Medicine, Cairo University, Cairo, Egypt; 2 Department of Pediatrics, City Clinic, Cairo, Egypt; 3 Pediatrics and Neonatology Department, Benha University, Benha, Egypt; 4 Pediatrics and Neonatology, Kasr El Aini Hospital, Cairo University, Cairo, Egypt; 5 Faculty of Postgraduate Childhood Studies, Ain Shams University, Cairo, Egypt; 6 Department of Pediatrics, Faculty of Medicine, Mansoura University, Mansoura, Egypt; 7 Pediatrics and Neonatology, Faculty of Medicine, Ain Shams University, Cairo, Egypt; 8 Pediatric Department, Faculty of Medicine, Assiut University, Assiut, Egypt; 9 Pediatric Consultant and Senior Consultant of the General Administration of Medical Affairs, Helwan University, Cairo, Egypt; 10 Military Medical Academy, Armed Forces College of Medicine, Cairo, Egypt; 11 Vaccination Centers in Egyptian Holding Company for Biological Products and Vaccines (VACSERA), Giza, Egypt; 12 Head of Pediatrics and Neonatology Department in Alexandria, New Medical Center (ANMC), Alexandria, Egypt; 13 Neonatology Department, Ain Shams University Hospital, Ain Shams University, Cairo, Egypt

**Keywords:** Vaccination, Immunization, Preterm, Low birth weight, Egypt, Premature, Vaccine, Vaccination schedule

## Abstract

**Background:**

Preterm birth has become increasingly common over the past several decades. The underdeveloped immune systems place preterm infants at an increased risk of infections, often vaccine-preventable, compared to full-term infants. Despite the importance of immunizing preterm infants, research on vaccine safety, immunogenicity, and effectiveness in this population remains limited. This consensus article seeks to provide clear, evidence-based recommendations to improve vaccine coverage and timely immunization in preterm infants in Egypt.

**Methods:**

A modified Delphi consensus approach was employed to develop evidence-based recommendations for preterm infant vaccination in Egypt. A panel of 18 experts, including pediatricians, neonatologists, and a pharmacist participated. The process covered 15 key vaccination topics, with 59 statements formulated based on a comprehensive literature review. A 5-point Likert scale was used to evaluate the statements, with ≥ 70% agreement threshold for consensus. Statements not meeting this threshold were revised and subjected to a second voting round. Final recommendations were established based on the achieved consensus.

**Results:**

Our consensus included 59 statements. In the first round of voting, 63 statements were evaluated, with the panel reaching a consensus on 54 statements. Nine statements fell below the 70% agreement threshold and required a second round of voting. A second round was conducted, including these nine statements along with one newly added statement. Ultimately, five statements achieved consensus, while five were removed.

**Conclusion:**

Establishing preterm vaccination guidelines is important for reducing mortality and morbidity in this high-risk population, especially in Egypt. This consensus emphasizes the need for timely immunization to minimize the risk of infections associated with delayed vaccination. Additionally, it identifies several areas requiring further research, ensuring that future updates can continue to refine and enhance immunization strategies for preterm infants.

**Clinical trial number:**

Not applicable.

**Supplementary Information:**

The online version contains supplementary material available at 10.1186/s12879-025-12243-0.

## Introduction

Preterm birth has become increasingly common over the past several decades, with global statistics showing that approximately one in nine infants worldwide is born prematurely [1]. The World Health Organization (WHO) categorizes preterm birth as any delivery that occurs prior to 37 completed weeks of gestation. This classification encompasses three key subgroups: births before 28 weeks are considered extremely preterm, those between 28 and 32 weeks are classified as very preterm, and births from 32 to 36 weeks are designated as moderate to late preterm. According to recent WHO data, an estimated 15 million babies are born prematurely each year, which is more than one in every ten live births worldwide. Complications related to preterm birth result in about 1 million deaths annually, while many survivors experience long-term challenges, including learning disabilities and visual or hearing impairments [2]. In Egypt, the prevalence of preterm births was estimated to be less than 10% in the general population [3].

Premature infants have underdeveloped immune systems, placing them at an increased risk of infections, not only during their initial neonatal period but also throughout their development [4]. Their vulnerability persists well beyond the newborn stage, with studies showing they are 2 to 3 times more likely to require hospital readmission due to infectious complications during infancy, childhood, and adolescent years [5–7].

Various procedures are implemented for preterm infant care, encompassing essential medical interventions provided in the Neonatal Intensive Care Unit (NICU), such as respiratory support and temperature regulation [8]. Despite advances in the NICU that have improved the survival of preterm infants, first-year mortality remains high, often due to delayed or incomplete immunization against vaccine-preventable diseases [9]. Africa and Asia bear the greatest burden of newborn deaths and complications, with developing nations experiencing significantly higher rates of neonatal mortality and morbidity compared to other regions. Premature birth, complications during delivery (such as birth asphyxia or trauma), neonatal infections, and congenital anomalies continue to be the primary causes of neonatal mortality [10]. Globally, an estimated three million infants die each year due to infections, with 2.8 million deaths occurring between one and 59 months of age, many of which could be prevented through timely immunization [11].

Vaccination plays a critical role in reducing mortality in this high-risk population. However, due to their immature immune systems, Preterm infants exhibit variable immune responses to vaccines [12].

Egypt’s healthcare system is primarily centralized under the Ministry of Health and Population (MOHP), which oversees neonatal care across public, university, and private hospitals [13]. The Egyptian health map demonstrates a wide distribution of neonatal care facilities across all 27 governorates, with the highest concentrations in Cairo and Alexandria. However, despite this distribution, there remains a substantial gap between need and capacity. Each year, about 500,000 infants require NICU admission, while the total number of NICU beds across MOHP and university hospitals does not exceed 4,000. Consequently, a significant proportion of critically ill newborns may be unable to access timely intensive care, reflecting the persistent shortage highlighted by the 2014 national statistics, which indicated that only 37.8% of the target NICUs have been achieved [13, 14].

According to Egypt’s national Expanded Programme on Immunization (EPI), all vaccines are provided free of charge under the supervision of MOHP. The schedule begins at birth with *Bacillus Calmette–Guérin* (BCG), oral polio vaccine (OPV), and hepatitis B virus (HBV) vaccine administered as “zero doses.” Immunizations at 2 months of age include DTwP-Hb-Hib (diphtheria, tetanus, whole-cell pertussis, HBV, and *Haemophilus influenzae* type b), OPV, and inactivated polio vaccine (IPV), with subsequent doses given at 4 and 6 months. OPV boosters are administered at 9, 12, and 18 months to maintain Egypt’s polio-free status. Booster doses of DTwP are also given at 18 months, while the measles, mumps, and rubella (MMR) vaccine is given at 12 and 18 months. Td and the polysaccharide bivalent meningococcal AC are provided at school age. Infants with a history of adverse events following pertussis-containing vaccines continue their vaccination schedule with the DT vaccine (diphtheria and tetanus only) [15].

The United Nations Children’s Fund (UNICEF) reports that Egypt’s EPI has reached more than 95% vaccination coverage. This has led to a significant reduction in vaccine-preventable diseases such as diphtheria, tetanus, pertussis, measles, and poliomyelitis [16].

The MOHP, supported by WHO and UNICEF, maintains high coverage through actual implementable steps, such as intensified immunization campaigns, improving cold-chain infrastructure, and actively monitoring for acute flaccid paralysis [16, 17].

As a result, Egypt has been polio-free since 2006 due to the consistent high coverage through biannual national immunization days and continuing environmental monitoring [17]. Moreover, it was removed from the list of Maternal and Neonatal Tetanus (MNT) endemic countries in 2007, and in 2023, the WHO declared that Egypt was measles-free [16].

Despite the importance of immunizing preterm infants, research on vaccine safety, immunogenicity, and effectiveness in this population remains limited. Many studies have small sample sizes and show variability in demographics, vaccine schedules, and inclusion criteria, making it challenging to establish definitive guidelines [9]. Evidence on long-term protection and vaccine effectiveness in preterm infants remains limited, especially in the Middle East and North Africa (MENA) region. Current guidelines recommend following the same chronological age-based vaccination schedule as full-term infants, with only a few specific exceptions, such as in HBV vaccination [18].

Several reasons contribute to the vaccination delays in preterm infants, including clinical instability, provider misconceptions about vaccine safety and efficacy, lack of clear guidelines, and the fear of adverse events [19–21]. Although preterm infants may have a weaker immune response to certain vaccines, evidence indicates that it is still sufficient to provide protection. Vaccines are generally safe for preterm and/or low birth weight infants, with safety profiles similar to those of full-term infants, though there may be a slight increase in the risk of apnoea, with or without bradycardia, within 48 h after immunization [22].

This article aims to establish the first Egyptian expert consensus on vaccinating preterm infants, focusing on safety, immunogenicity, and effectiveness. Currently, there is no unified vaccination schedule or national recommendations specifically tailored for preterm infants in Egypt, representing a critical gap in clinical practice. By synthesizing current evidence and incorporating local perspectives, this consensus seeks to provide clear, evidence-based recommendations to improve vaccine coverage and timely immunization in preterm infants. The publication of these recommendations will support upgrading vaccination practices and contribute to reducing the risk of infections in this high-risk population.

## Methods

**Fig. 1:**
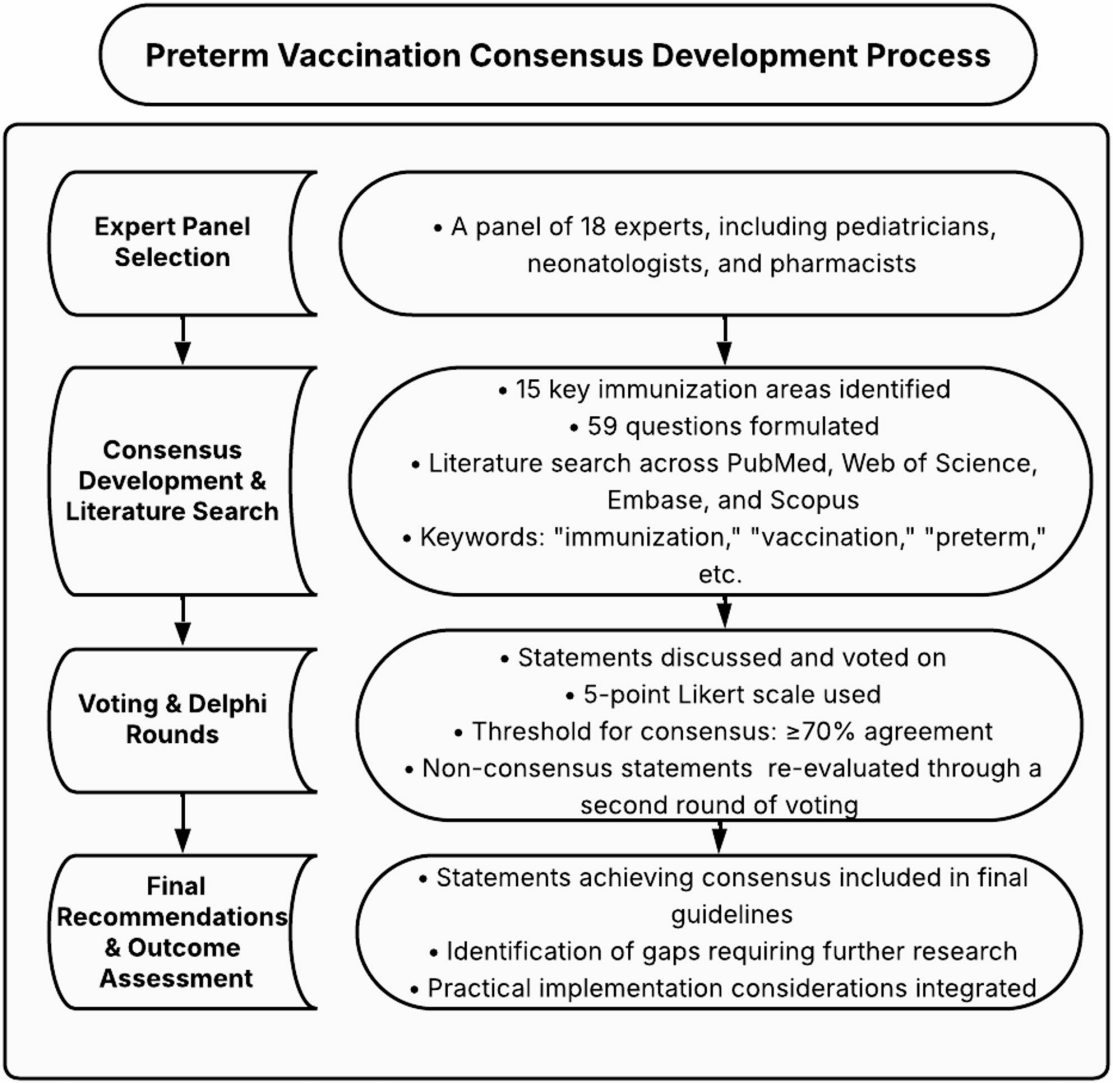
Preterm vaccination consensus development process

This consensus was made by a modified Delphi consensus approach, which we employed to develop evidence-based recommendations for preterm infant vaccination. This consensus focuses on establishing practical recommendations for vaccination practices in preterm infants in Egypt. The consensus covers 15 distinct vaccination topics, ranging from routine immunizations to specific vaccines relevant to preterm infants. It also addresses indirect protection strategies through family vaccination, making it comprehensive for both direct and indirect immunization approaches. The main phases of the modified Delphi approach are shown in Fig. 1.

### Expert panel selection

The multidisciplinary panel consisted of 18 experts, including pediatricians, neonatologists, and a pharmacist, all with prior experience in pediatric immunization; these panel members are also experienced in preterm infant care and vaccination. All panel members who participated in the modified Delphi process are co-authors of this manuscript. Each expert contributed equally to the evidence synthesis, discussions, and development of the final recommendations. The voting process was conducted in a blinded manner to ensure objectivity and minimize potential bias.

### Consensus development process

The consensus is structured on distinct parts that cover different aspects regarding preterm vaccination. The consensus covers 15 key areas of immunization in preterm infants, including 59 questions (Table S1), addressing critical aspects such as general vaccination challenges, adverse events, strategies for indirect protection through family immunization, and specific recommendations for individual vaccines, which includes BCG, HBV, pneumococcal, rotavirus, seasonal influenza, DTP-containing vaccine, meningococcal, *Haemophilus influenzae* type b (Hib), poliovirus, MMR, and chickenpox. As well as recommendations for the role of mAB in preventing Respiratory Syncytial Virus (RSV) disease. After developing the questions, they were covered after a comprehensive literature search till Jan 2025 through PubMed, Web of Science, Embase, and Scopus. This search explored the questions associated with vaccinations in preterm infants using keywords such as “immunization,” “vaccination,” “preterm,” “low birth weight,” and “premature infants.” Further manual search using free text terms was employed to allocate further related studies. After completing the search, the statement of each question was made.

### Voting process

The voting process followed a systematic approach, beginning with an in-person round where each statement was presented to the panel for discussion and subsequent voting. For each statement, experts evaluated their level of agreement using a 5-point Likert scale, ranging from strongly disagree to strongly agree. The responses were analyzed using Microsoft Excel software to calculate an agreement score for each statement. A threshold of ≥ 70% agreement (combining “strongly agree” and “agree” responses) was established to determine positive consensus. Statements that did not meet this threshold were revised and subjected to a second round of voting through an online survey, following the same modified Delphi approach.

### Outcome assessment

The outcome assessment involved formulating final recommendations based on statements that achieved consensus while clearly identifying areas that either required additional research or lacked consensus among the panel. The process also ensured that practical implementation considerations were carefully integrated into the final recommendations, creating a comprehensive and actionable set of guidelines.

## Results

Our consensus included 59 statements distributed across 15 sectors, addressing various aspects of immunization in preterm infants. In the first round of voting, 63 statements were evaluated, with the panel reaching consensus on 54 statements. Nine statements fell below the 70% agreement threshold and required a second round of voting. After rephrasing, further discussion, and modifications, a second round was conducted, including these nine statements along with one newly added statement. Ultimately, five statements achieved consensus, while five statements were removed as they didn’t reach a consensus.

The voting results demonstrated very strong agreement (≥ 90%) in 42 (71.2%) of the statements and strong agreement (≥ 70% but < 90%) in 17 (28.8%) of the statements. A summary of the consensus statements and Vaccine recommendations is presented in Table 1.

**Table 1:** Summary of the consensus statements on vaccine recommendations

Vaccine	Recommendations	Recommended at Birth
RSV Disease	Extended half-life mAbs (Nirsevimab) should be administered from birth for infants born during the RSV season (preferably prior to discharge or within one week) and up to 24 months of age who remain vulnerable to severe RSV disease through their second RSV season. For others born outside the season, it should be administered ideally prior to the RSV season.	**Yes** (if born during RSV season)
Polio Vaccine	IPV→ 4 doses at 2, 4, 6, and 18 months. (Preferably within the combination vaccine) OPV→ 2 doses at 4 and 6 months.	No
HBV Vaccine	**< 2 kg**: -Positive / Unknown: HBV + HBIG -Negative: HBV **≥ 2 kg**: -Positive: HBV + HBIG -Unknown: HBV (maternal serology, consider HBIG within 1 week if mother positive) -Negative: HBV If HBIG given → do serology at 9–12 months All infants should receive HBV-containing combination vaccines at 2, 4, and 6 months.	**Yes** (depending on maternal HBsAg status and weight)
BCG Vaccine	Single dose for those ≥ 34 weeks of gestation and ≥ 2 kg weight (Contraindicated in infants with known or suspected immunodeficiency, including those with a family history of primary immunodeficiency)	**Yes** (if ≥ 34 weeks & ≥2000 g)
Diphtheria, Tetanus, Acellular Pertussis. Hepatitis B, Inactivated Polio, and Hib	3 primary doses at 2,4,6 months and a booster at 12–18 months	No
Rotavirus Vaccine	The first dose at 6–15 weeks of chronological age. The following doses are as per the schedule of available formulation, with a minimum interval of 1 month apart.	No
Pneumococcal Vaccine	3 primary doses of PCV at 2,4,6 months and a booster at 12–15 months 2 doses of PPSV23 at 4 & 9 years	No
MenACWY vaccine	Should be initiated during the first year of life, as early as the recommended schedule of the available pharmaceutical formulations	No
MenB vaccine	Three doses: the first dose as early as 2 months of age, with a minimum 2-month interval between the first and second doses. The third dose should be given at or after 12 months of age, with a minimum 6-month interval between the second and third doses.	No
Measles, Mumps, and Rubella Vaccine	2 doses starting at 12 months (minimum 3 months interval)	No
Varicella Vaccine	2 doses starting at 12 months (minimum 3 months interval)	No
Hepatitis A Vaccine	2 doses starting at 12 months (minimum 6 months interval)	No
Seasonal Influenza Vaccine	Infants ≥ 6 months should receive two doses, one month apart in the first year followed by one dose annually in subsequent years.	No

## Recommendations and discussion

### Sector (1): Vaccination challenges and considerations for preterm infants

#### Chronological age vs. corrected age for vaccination

The panel addressed several challenges regarding the vaccination of preterm infants, emphasizing the importance of using chronological age rather than corrected age to determine vaccination timing (Statement 1.1; Table S2). Although controversies exist around the right age suitable for preterm immunization, much evidence supports the chronological age as their term counterparts [23]. In fact, the delay in vaccination is likely to extend the period of infection susceptibility, thereby increasing the risk of infection [24]. The protective effect of current vaccination relies on the production of neutralizing antibodies. Thus, to promote protective effects as soon as possible, preterm infants need to be immunized at the same chronological age as their term peers [24]. A recent review by Flannery et al. emphasized that most vaccines are given based on chronological age, irrespective of birth weight or gestational age. The exception to this is the HBV vaccine, which is given according to maternal hepatitis B surface antigen status (HBsAg) and birth weight [25].

Despite the current recommendations regarding the time of vaccination in preterm infants to be on the same schedule as full-term infants, many studies have proved unjustified delays, though these vaccinations are well tolerated in this population [26, 27]. The panel strongly recommended that preterm infants should follow the same vaccination schedule as term infants, regardless of corrected age (Statement 1.2; Table S2). Chiappini et al. confirmed that the scheduled vaccines are safe and well-tolerated, showing no distinctions between preterm and full-term infants. Additionally, live attenuated vaccines are also deemed safe [28]. Sadeck et al. recently proposed that vaccinations for preterm infants should follow their chronological age, using the same schedule as that for term infants, with limited exceptions and without adjustments for gestational age or birth weight [9].

#### Postponing vaccination in unstable preterm infants

The panel unanimously recommends that vaccination should be postponed in preterm infants presenting with unstable hemodynamic conditions, sepsis, infectious diseases, or neuro-metabolic disorders (Statement 1.3; Table S2). A recent study conducted in Brazil supported this statement, highlighting that in preterm infants with unstable hemodynamic conditions, the primary focus is stabilizing the patient before administering the vaccination. For infants with sepsis or infectious diseases, vaccination should be postponed until they have fully recovered to ensure their immune system is capable of achieving an adequate response [29].

#### Preferred intramuscular injection site

The panel agreed that Intramuscular (IM) vaccines can be safely administered to preterm infants at their chronological age, provided their weight ≥ 2000 g (Statement 1.4; Table S2).

Regarding the suitable muscle for the IM vaccination, they preferred the vaccination in the vastus lateralis muscle in infants due to its larger muscle mass (Statement 1.5; Table S2). In a randomized controlled trial (RCT) on Chinese infants, the results demonstrated equal safety and immunogenicity for the Hib vaccine in the vastus lateralis concerning the local and systematic reactions after the first two injections, with a notable reduction in the systematic reaction in the vastus lateralis following the third injection [30]. In another study involving 185 healthy Turkish infants (mean age = 4 months), they found that the duration of crying was shorter in those who received the vastus lateralis vaccination compared to those who were vaccinated in the deltoid region [31]. There is limited data on the preferred vaccination site for preterm infants. However, Rishovd et al. emphasize that the vastus lateralis muscle is the preferred site for both preterm and full-term infants [32]. This is due to its larger muscle mass and a reduced risk of complications.

Meanwhile, 89% of the panel disagreed with changing the vaccination site for very low birth weight infants (< 1500 g) (Statement 1.6; Table S2). While there is no definitive optimal vaccine site for Very-Low-Birth-Weight (VLBW), there is generally a preference to adhere to the same immunization settings as for those closer to term weight. The vastus lateralis muscle remains the highly recommended site for vaccine administration in this age group [33, 34].

#### Needle length for intramuscular vaccination

Selecting the correct needle length for intramuscular vaccination in preterm infants is critical. This choice should consider the available muscle mass, with the needle shorter than the standard 7/8 inch to 1 inch used for full-term infants. Because PT and LBW infants have limited muscle mass, shorter needles (≤ 5/8 inch) may be more suitable to ensure safe and effective intramuscular injection into the anterolateral thigh for HBV vaccination [35]. The panel validated shorter needles (< 16 mm) for intramuscular vaccinations in preterm infants to accommodate reduced muscle mass (Statement 1.7; Table S2). Kempe A. recommends using a 23 or 25-gauge, 16 mm needle for preterm and very small infants [36]. It’s essential to administer the injection slowly to minimize pain and reduce the risk of muscle trauma.

#### Avoiding vaccination at compromised skin sites

94% of the panel chose to avoid vaccination in areas of skin lesions, bruising, or scarring in preterm infants (Statement 1.8; Table S2). This is because such conditions can complicate the vaccination process and increase the risk of adverse reactions, such as eczema vaccinatum, particularly in individuals with pre-existing skin diseases [37]. There is limited data on vaccinating preterm infants at sites with skin lesions, bruising, or inflammation, highlighting the need for further research to guide best practices in such cases.

#### Live attenuated vaccines in hospital settings

The panel advises against administering live virus vaccines in hospital settings that include immunocompromised individuals (Statement 1.9; Table S2). Rotavirus is a common cause of acute viral gastroenteritis in infants. Both Canadian and American guidelines recommend that immunization with the rotavirus vaccine be administered to infants, particularly preterm infants, only upon their discharge from the hospital [38–40]. While 83% of the members agreed that rotavirus vaccines should not be used in hospital environments, some expressed reservations. Sicarda et al. thoroughly discussed the safety of rotavirus in preterm infants and neonates in the ICU. Their finding indicated that the viral shedding occurred in nearly all vaccinated infants, mainly within the first week after the first dose. Still, instances of transmission were rare and only occurred within households, with no reported cases in the NICU. The adverse events following vaccination were mild to moderate, affecting 10–60% of infants. Notably, the extremely premature or infants who had gastrointestinal failures requiring surgery experienced more severe reactions [41]. Chiu et al. found that in certain immunocompromised infants (infants infected with or exposed to HIV), rotavirus vaccination didn’t show an increase in adverse events. However, case reports highlighted an elevated risk of adverse events in infants with severe combined immunodeficiency. Despite the vaccine being proven to be well-tolerated and effective, there are ongoing controversies surrounding the safety of the rotavirus vaccine in preterm and immunocompromised infants [42].

Concerns regarding the transmissibility of live attenuated vaccines largely stemmed from the OPVs [43]. Although wild polio cases are decreasing, vaccine-associated paralytic poliomyelitis (VAPP) has become a significant issue that impacts both those who received the vaccine and individuals who came into contact with them [43]. According to recent data from the WHO and the Centers for Disease Control and Prevention (CDC), global polio cases have decreased by more than 99% since 1988 through the Global Polio Eradication Initiative. The WHO Regions of the Americas (1994), Western Pacific (2000), and Europe (2002) have all been certified polio-free, followed by the South-East Asia Region in 2014 and the African Region in 2020. VAPP has been virtually eliminated in countries that exclusively use the IPV [44, 45].

Given this risk, there was an agreement that the OPV should not be used in these environments due to the risk of transmission. Although live vaccinations were generally safe and effective in healthy infants, some severe reactions and vaccine-related infections have been observed, especially in patients with conditions such as HIV, leukemia, immunosuppressed inflammatory diseases, and organ transplant patients [46].

According to a review article conducted in 2021 for infection prevention and control in the NICU, vaccines containing inactivated components do not carry any infection risk to other NICU patients [47]. The risk of transmission from live-attenuated vaccine strains is minimal; however, vaccines like rotavirus and live-attenuated influenza vaccine (LAIV) have a theoretical possibility of viral shedding. Therefore, NICU units may consider applying contact precautions (e.g., for rotavirus) for up to two weeks after vaccine administration to minimize any potential cross-infection risk [47].

### Sector (2): BCG vaccine

A total of 1.25 million people died from *tuberculosis* globally, with around 10.8 million people developing the disease in 2023 [48]. *tuberculosis* has likely re-emerged as the leading cause of death worldwide from a single infectious agent [48]. BCG, the sole approved vaccine for *tuberculosis* prevention, is the most widely administered vaccine globally [49].

In Egypt, *tuberculosis* has significantly decreased over the last two decades [50, 51]. This is attributed to the BCG vaccination coverage rate, which reaches 98% [52]. A recent systematic review and meta-analysis estimated the overall *tuberculosis* prevalence at 8.7 per 100,000 population (95% CI: 5.8–12.4; I² = 92.7%), with an incidence rate of 9.1 per 100,000 population (95% CI: 6.7–14.9; I² = 95.5%) [51].

In full-term infants, the BCG vaccine is typically administered shortly after birth, helping to lower the risk of developing *tuberculosis* disease and reducing *tuberculosis* -related mortality during childhood [53]. However, BCG is commonly delayed in preterm infants as well as low birth weight due to uncertainty about safety and immunogenicity [53].

An early study by Thayyil-Sudhan et al. was conducted on 62 preterm infants (gestational age < 35 weeks) who received BCG vaccines early. They proved the safety and efficacy of BCG vaccination within days after birth for premature babies [54]. In another randomized study by Camargos et al., they compared preterm infants to a control group of full-term infants and recommended administering the BCG vaccine to preterm infants upon their discharge from the NICU in order to reduce morbidity and mortality in infants at risk of *tuberculosis* infection and to increase BCG vaccination coverage rates [55].

A systematic review by Badurdeen et al. concluded that BCG vaccination within the first 7 days of life in healthy preterm neonates (born at >30 weeks or weighing >1.5 kg) did not increase the risk of adverse events or infant mortality compared with later vaccination in clinically stable infants who were preterm and/or low birth weight. The meta-analysis further found no significant differences in tuberculin skin test conversion or scar formation [53]. Furthermore, the WHO recommends administering the BCG vaccination at birth for healthy preterm infants born between 32 and 36 weeks of gestation, as it has been shown to be both safe and effective [56]. However, evidence on the safety and effectiveness of BCG vaccination in very preterm and extremely preterm infants remains scarce.

The minimum gestational age for safely administering the BCG vaccine at birth is not well-established [57]. The panel strongly recommended postponing the BCG vaccination in preterm infants under 34 weeks until they reach 34 weeks of gestational age (Statement 2.1; Table S3). The panel also clarified that BCG vaccination should be administered at birth for clinically stable preterm infants born at ≥ 34 weeks gestational age and weighing ≥ 2000 g. This is consistent with current recommendations reported by Sioriki et al. and the Immunisation Advisory Centre in New Zealand [1, 58]. On the other hand, a study on moderate preterm infants (31–33 weeks) who were vaccinated with BCG at birth found that the BCG vaccine showed high safety and immunogenicity. However, no significant differences were observed compared to preterm infants who were administered BCG at 34 weeks [57].

Reports from developing countries indicate that they often delay BCG vaccination [59, 60]. This postponement is due to various reasons, including lower socioeconomic status, parental awareness, and fear of adverse events [61]. The panel validated postponing the BCG vaccination in infants < 2000 g (Statement 2.2; Table S3).

A retrospective study in Guinea-Bissau showed that the percentage of low-birth-weight infants who had not received the BCG vaccine was 1.5 to 3 times higher than that of normal-weight infants up to 4 months old. They also concluded that administering BCG vaccination at birth is more beneficial for the survival of low-birth-weight preterm infants than postponing it [62]. Another study in India indicated that infants with low birth weight < 2,000 g had higher odds of experiencing delays in receiving the BCG vaccine (adjusted odds ratio [aOR] 2.33, 95% CI 1.89, 2.89) [63].

In a case report, a woman with refractory Crohn’s disease received infliximab during her pregnancy and gave birth to a healthy baby. The infant received the BCG vaccine at three months but later became ill and died at 4.5 months. The cause of death was identified as disseminated BCG, which is a rare complication related to the vaccine. Therefore, it is advisable to delay BCG vaccination for at least 7 months for mothers undergoing treatment with immunosuppressive IgG1 antibodies or anti-TNF therapies [64]. Moreover, in a clinical study in the Czech Republic on infants ≥ 12 months previously exposed to infliximab in utero, 15 infants received the BCG vaccine within one week of birth. Three children experienced large local skin reactions, with one case complicated by axillary lymphadenopathy. All reactions resolved without requiring antituberculosis treatment, and no further complications were reported following BCG administration [65]. Ling and Koren concluded that the standard vaccinations are typically well tolerated in infants who were exposed to biologics prenatally to reduce the infection risk. However, live vaccinations should be delayed during the first six months to lower the risk of dissemination [66]. Although evidence is limited for preterm infants of mothers with an unknown HIV status or those born to HIV-positive mothers, the WHO recommends that they should receive the BCG vaccine, as the benefits are considered to outweigh the potential risks. Research on its use in preterm infants of mothers taking immunosuppressants remains scarce. However, if a mother is HIV-positive and her baby’s HIV status is unclear, vaccination is recommended unless there are clinical signs of HIV infection, regardless of whether the mother is undergoing antiretroviral therapy [56]. Most of the panel agreed that BCG vaccination guidelines should be modified for preterm infants born to mothers who used immunosuppressants (Statement 2.3; Table S3).

Given the inadequacy of data regarding the BCG vaccination’s effectiveness in preventing respiratory infections and neonatal sepsis, the panel advised not to administer BCG earlier than the recommended age and weight thresholds to improve their resistance to these conditions (Statement 2.4; Table S3). In a retrospective study by de Castro et al. in Spain, researchers found that BCG vaccination at birth could reduce hospitalizations due to respiratory infections and sepsis unrelated to *tuberculosis* through heterologous protection [67].

### Co-administration with hepatitis B vaccine

BCG is a live-attenuated vaccine administered intradermally, whereas the hepatitis B vaccine is an inactivated vaccine administered intramuscularly. Because the vaccines use different routes of administration, no clinically meaningful immune interference has been observed when both are administered at birth. This approach is common practice in many national immunization programs. For clinically stable preterm infants who meet BCG eligibility criteria (≥ 34 weeks gestation and ≥ 2000 g), both vaccines may be given at the same visit. Evidence on simultaneous administration in extremely preterm or very low birth weight infants is minimal, and more research is necessary [68].

### Sector (3): HBV vaccine

HBV infection represents a major global health challenge, causing high mortality rates and substantial burdens [69]. In Egypt, the population prevalence rate for HBV in this study was 1.4% (95% CI: 1.2–1.6%) in adults aged 15–59, based on national survey data [70]. HBV infections in infants primarily occur through vertical transmission from mother to child [71].

Preterm and low birth weight infants (< 2 kg) have an elevated risk of perinatal transmission and show a poorer vaccine response compared to term infants, even when standard post-exposure prophylactic measures are administered [72]. The panel strongly recommended that preterm infants with HBsAg-unknown mothers be treated as HBsAg-positive and receive hepatitis B immunoglobulin (HBIG) as well as HBV vaccine at birth, followed by three additional doses at 2, 4, and 6 months of age. The discussion emphasized the risks of chronic infection and the need for clear maternal screening (Statement 3.1; Table S4).

The current guidelines for HBV from the Advisory Committee on Immunization Practices (ACIP) and the American Academy of Pediatrics (AAP) do not provide specific recommendations for infants born before 37 weeks of gestation. However, the ACIP advises that for infants with a birth weight of less than 2000 g, the first dose of the vaccine should be delayed until the infant is 1 month old or until they are discharged from the hospital if their mothers are HBsAg-negative [73, 74]. The Immunization Advisory Center in New Zealand recommends that preterm infants born to HBsAg-negative mothers and/or weighing less than 2000 g are expected to be adequately protected against HBV after receiving three doses of the vaccine, starting at 6 weeks of age [58].

The CDC advises that all infants, regardless of the HBsAg status of their mothers, should receive their initial dose of the HBV vaccine within 24 h of delivery [75]. Likewise, in China, the National Immunization Program’s Childhood Immunization Procedures and Instructions state that infants weighing under 2000 g, born to mothers with HBsAg positive or unknown status, should receive the first dose of the HBV as soon as possible after birth [76]. Likewise, our panel strongly recommended that preterm infants of mothers with unknown HBsAg status be managed as HBsAg-positive, receiving HBIG and the HBV within 12 h of birth to ensure timely protection against infection (Statement 3.2; Table S4).

The infant’s immune system immaturity and chronological age are the primary factors determining the immune response to HBV. The majority of the panel agreed that infants weighing less than 2,000 g or born before 34 weeks should follow an adjusted HBV schedule based on their birth weight and clinical stability (Statement 3.3; Table S4).

Some studies suggest implementing a three-dose strategy may yield a reduced antibody response to HBV in VLBW infants who are born to HBsAg-negative mothers [74, 77], while other findings support a four-dose strategy [78]. In contrast to full-term infants, preterm infants, particularly those with VLBW who are exposed to HBV, may face a heightened risk of contracting HBV infection due to their diminished immunological response to existing vaccination protocols [79].

Sadeck and Ramos demonstrated that preterm infants with a gestational age of < 34 weeks were less likely to respond to the three-dose vaccination schedule. They recommended that preterm infants with a birth weight of ≤ 1500 g, who have a lower antibody response, should have their anti-hepatitis B titers monitored at nine months of age, or that a vaccination schedule should be adjusted to a four-dose vaccination schedule [80].

The panel strongly agreed that serology testing for HBsAg and anti-HBs should be conducted at 9 to 12 months of age for preterm infants born to HBsAg-positive or unknown mothers (Statement 3.4; Table S4). This recommendation is also supported by the ACIP [73]. Testing should be delayed until at least nine months of age to prevent false detection of passive anti-HBs from HBIG given at birth and to improve the chances of detecting late HBV infection [73].

Combination vaccines are routinely given to premature infants and have several advantages, including minimizing the number of injections and enhancing adherence to immunization schedules. The Hexavalent combination vaccine, which is often administered concurrently with pneumococcal vaccines, was shown to be generally well-tolerated [81]. HBV is either recommended at birth or as part of these combination vaccines. The panel recommended that both HBV combination vaccines and separately administered HBVs are safe for preterm infants, with an agreement of 89% (Statement 3.5; Table S4). Moreover, several studies have demonstrated that administering additional doses of the HBV vaccine does not have a negative impact on the infant [82, 83]. While subjects who received an extra dose exhibited higher anti-HBs antibody titers, these elevated levels are not considered clinically meaningful [82].

### Sector (4): RSV disease

Worldwide, acute lower respiratory tract infections (ALRIs), particularly bronchiolitis, are among the leading causes of childhood illness, death, and hospitalization. RSV is one of the most frequent viral triggers of childhood ALRI. Nearly 90% of children are infected with the RSV by the time they reach two years of age [84].

Preterm infants (gestational age < 37 weeks) with chronic lung disease of prematurity/BPD face a higher risk of hospitalization due to RSV-associated respiratory illness. They also tend to experience longer hospital stays for respiratory infections and are more likely to require specialized care compared to other children [84].

Currently, there is no RSV vaccine approved for direct use in infants. Developing an RSV vaccine specifically for infants has been particularly challenging for several reasons [85].

There are two RSV-specific monoclonal antibody (mAb) categories that can provide passive protection to infants: extended half-life mAbs, such as Nirsevimab, and the short-acting mAb- Palivizumab [85].

Accordingly, the panel strongly agreed on administering the mAb during or before the RSV season in preterm infants for direct protection from RSV disease (Statement 4.1; Table S5).

Palivizumab is approved for prophylaxis in specific “high-risk” groups, such as infants with congenital heart disease, bronchopulmonary dysplasia, or prematurity. It is administered as monthly intramuscular injections throughout the RSV season, usually totaling five doses. However, medical guidelines in some countries may further specify eligibility to a limited part of the birth cohort [86, 87].

Nirsevimab has been designed for the protection of all infants against RSV lower respiratory tract disease, with a single IM injection. It should be administered at birth for infants born during the RSV season (preferably before discharge or within the first week of life) and up to 24 months of age who remain vulnerable to severe RSV disease through their second RSV season. For infants born outside the RSV season, it should be administered ideally before the RSV season [88–90].

Nirsevimab is favored over palivizumab because of its markedly higher potency, showing more than 50-fold greater binding affinity and neutralizing activity against both RSV-A and RSV-B strains [91], Moreover, its mechanism of action allows for rapid and direct protection with a single intramuscular dose throughout the RSV season [85]. The panel unanimously favored Nirsevimab over palivizumab for RSV prevention in preterm infants born before or during the RSV season (Statement 4.2; Table S5).

### Sector (5): Pneumococcal vaccine

*Streptococcus pneumoniae* is a human pathogen that accounts for most bacterial pneumonia cases and invasive pneumococcal diseases, posing considerable morbidity and mortality risks [92]. Conjugate vaccines aimed at the pneumococcal capsule have significantly decreased the occurrence of these invasive diseases [93]. Preterm infants face a heightened risk for vaccine-preventable diseases, presenting a 2.5 to five-fold increased risk of experiencing severe invasive pneumococcal disease [23].

The panel strongly recommended that preterm infants receive four doses of pneumococcal conjugate vaccine (PCV) following the standard schedule at 2, 4, 6, and 12–15 months, and this is consistent with the Australian guidelines as well as the CDC (Statement 5.1; Table S6) [94–96]. An RCT study by Kent et al. assessed the safety of the PCV13 conjugate vaccine in preterm infants (< 35 weeks), comparing three vaccination schedules: reduced (2 and 4 months), accelerated (2, 3, and 4 months), and extended (2, 4, and 6 months). Results showed no significant differences in local or systemic adverse events across schedules. In total, 77 serious adverse events were reported, including two deaths [97]. Martinón-Torres et al. demonstrated that a four-dose regimen of PCV13, administered at 2, 3, 4, and 12 months, was immunogenic and well tolerated in both preterm and term infants. Although immune responses were generally lower in preterm infants, the majority in both groups achieved pneumococcal serotype-specific IgG antibody concentrations exceeding the WHO-defined threshold for protection, along with functional antibody responses [98].

The panel strongly recommended that preterm infants receive additional pneumococcal vaccine doses beyond the age of 5 under certain conditions (e.g., immunocompromised status and chronic medical conditions) (Statement 5.2; Table S6). A booster significantly improves antibody concentrations in preterm infants, regardless of the primary immunization schedule [97].

The panel additionally advised that preterm infants should receive two doses of the 23-valent pneumococcal polysaccharide vaccine (23vPPV) at ages 4 and 9 years (Statement 5.3; Table S6). This statement is in line with the existing Australian immunization schedule for the 23 V PPV [35, 95].

Although preterm infants are more likely to exhibit weaker responses to conjugate vaccines, these vaccines have been proven to be safe and well tolerated, with minimal local and systemic adverse events. They are recommended for all children, including preterm infants, provided the infants clinical condition allows for vaccination [35, 99].

### Sector (6): Rotavirus vaccine

Rotavirus is considered a prevalent cause of severe gastroenteritis in children < 5 years old; it accounts for an estimated 185,400 deaths worldwide annually [100, 101]. Preterm neonates are more prone to develop severe rotavirus infection [102]. Rotavirus prevalence in Egypt varies widely, ranging from 11% to 76.9% across studies, influenced by regional, seasonal, demographic, and methodological differences [103, 104].

The panel contraindicated administering the oral live attenuated rotavirus vaccine in preterm infants with suspected immunodeficiency or whose mothers used biologicals during pregnancy (Statement 6.1; Table S7). The current guidelines recommend against administering rotavirus to infants with in-utero exposure to biologics until they are at least 6 to 12 months old [105, 106].

In the case of VLBW infants (< 1500 g), the panel did not recommend administering the oral rotavirus vaccine to preterm infants weighing less than 1500 g or before hospital discharge. A retrospective study by Dahl et al. on USA neonates showed a significant reduction in acute gastroenteritis hospitalizations following rotavirus vaccination, which was similarly effective in LBW and VLBW infants compared to those with normal body weight [107]. The panel’s precautionary approach may be due to the limited data on hospitalized VLBW infants (< 1500 g) and the fear of nosocomial transmission in the NICU setting. Further research, particularly in our region, is needed to validate this statement.

The panel recommended administering the first dose of the rotavirus vaccine to healthy preterm infants between 6 and 15 weeks of chronological age (Statement 6.2; Table S7). Our statement is based on the WHO and the Swedish Public Health Agency, which recommend vaccination of preterm infants with the first dose of the RV between 6 and 12 weeks of age [108, 109]. This schedule is essential to provide infants with vital protection during the crucial early months of their lives.

The panel determined that the oral live attenuated rotavirus vaccine should not be routinely administered to hospitalized age-eligible preterm infants (Statement 6.3; Table S7). A multicenter observational study by Costantino et al. examined the safety of rotavirus vaccination in preterm infants ≥ 28 weeks across NICUs in Italy, confirming that rotavirus vaccination has no significant adverse events [110]. The Swedish national guidelines mandate that preterm infants admitted to the neonatal ward be vaccinated with the first dose against rotavirus infection when hospitalized and age-eligible [109]. The panel’s precautionary approach may be due to the limited data on hospitalized preterm infants and the fear of nosocomial transmission in the NICU setting. Further research, particularly in our region, is needed to validate this statement.

In several countries, the rotavirus vaccine has been contraindicated in hospitalized infants because of the possible risk of dissemination of the vaccine virus within the NICU. However, several more recent studies have not confirmed this risk. Since the rotavirus vaccine has a maximum age limit for the first dose, this restriction often prevents timely administration [111].

The panel unanimously recommends that preterm infants who are discharged at the time of rotavirus vaccination should follow the same immunization schedule as full-term infants (Statement 6.4; Table S7). Several studies clearly demonstrated that the safety and tolerability of scheduled vaccines, including rotavirus vaccination, are comparable between preterm and full-term infants [38, 102, 112].

### Sector (7): Seasonal influenza vaccine

Children under 5 years old, particularly those under 2, as well as children of any age with specific chronic health problems, are at an elevated risk of experiencing potentially severe complications associated with influenza [113].

Premature infants, especially those with chronic lung disease, have a higher risk of influenza-related hospitalization compared to full-term infants. Influenza vaccination is recommended at six months of chronological age, as younger infants may have reduced immunogenicity due to maternal antibody interference. Between 6 and 17 months, the immune response of preterm infants to influenza vaccines is comparable to that of term infants, with no records of a higher incidence of adverse events in this population [9, 23].

The ACIP recommends annual influenza vaccination for all persons aged ≥ 6 months who do not have contraindications [114]. To achieve optimal protection, all children between 6 months and 8 years must receive two doses separated by at least 4 weeks if receiving the vaccine for the first time. Persons aged ≥ 9 years need only one dose [114]. The panel strongly recommended that all infants, including the preterm aged 6 months or older, receive two doses of the influenza vaccine, administered one month apart in the first year, followed by a single annual dose in subsequent years (Statement 7.1; Table S8).

### Sector (8): DTP-containing vaccine

Preterm infants are especially vulnerable to vaccine-preventable diseases such as pertussis, pneumonia, and meningitis, and protection through vaccination is desirable at the earliest age possible [25, 115].

Combined vaccines have contributed to an increase in adherence and VCR by reducing the number of injections, a better safety profile, the flexibility to be co-administered with other vaccines, as well as the reduction of costs [116–118]. This made many countries decisively transition from wP vaccines to aP vaccines, especially the Hexavalent formulation (DTaP-IPV-HB-Hib), for those reasons [119, 120]. The panel agreed that higher-valency vaccines, such as Hexavalent (DTaP-IPV-HB-Hib) formulations, are preferred over lower-valency or separate vaccines to minimize the number of injections required for preterm infants (Statement 8.3; Table S9).

In many studies, Hexavalent formulations (DTaP-IPV-HB-Hib) have demonstrated robust immune responses for all antigens post-primary series and post-booster in preterm infants with a reassuring safety profile [25, 121, 122], and can be safely co-administered with other vaccines such as pneumococcal vaccines, measles, mumps, rubella vaccines, varicella vaccines, rotavirus vaccines, or meningococcal vaccines [117].

Preterm infants should receive three doses of DTaP-containing vaccines at 2, 4, and 6 months of age, based on their chronological age, considering the hemodynamic stability, followed by a booster dose at 12–18 months, to ensure continued protection [25]. The panel strongly recommended that preterm infants receive three doses of diphtheria, tetanus, acellular pertussis, IPV, Hib, and HepB vaccine at 2, 4, and 6 months of age, based on their chronological age, followed by a booster dose at 12–18 months, to ensure continued protection (Statement 8.4, 8.5; Table S9).

### Sector (9): Meningococcal vaccine

Neonatal meningitis remains a significant concern in neonatology and pediatric infectious diseases, with an incidence ranging from 0.8 to 6.1 per 1,000 live births, high case fatality rates, and frequent neurological complications. Premature birth is one of the most important risk factors for developing meningitis in the neonatal period [123].

Six serogroups (A, B, C, W, X, and Y) are linked to invasive infection, with A and W being the most common in the Middle East [124].

Polysaccharide vaccines have several limitations because they do not provide immune memory against the capsules of pathogens under 2 years old [125–127]. Moreover, they provide only short-term immunity lasting 3 to 5 years after vaccination and do not reduce mucosal carriage or promote herd immunity. On the other hand, conjugate vaccines elicit a robust immune memory response, making them effective for toddlers under 2 years of age and older. The WHO recommends conjugate vaccines over polysaccharide vaccines because they offer herd protection and have greater immunogenicity, especially in early childhood children [128].

Considering the polysaccharide vaccines’ limitations, the panel recommends that polysaccharide vaccines should not be given to children under 2 years of age, and any infants, regardless of gestational age, should receive a conjugate meningococcal vaccine according to the guidelines in each product’s insert leaflet (Statement 9.1; Table SS10).

The MenACWY vaccine provides direct and indirect protection through herd immunity against four serogroups [129]. Research indicates that MenACWY-TT has strong immunogenicity across age groups and is well-tolerated with an acceptable safety profile [130]. The panel strongly recommended that MenACWY should be routinely administered to preterm infants (Statement 9.2; Table S10). Despite the paucity of studies on MenACWY efficacy in preterm infants, solid evidence supports administering MenACWY-TT to infants from 6 weeks old. MenACWY-TT has proven to be safe and well tolerated, inducing an immune response after both the initial dose and the booster and maintaining a persistent immune response [130].

The panel recommended receiving a two-dose primary series of the meningococcal conjugate vaccine (MenACWY) for preterm infants as early as the recommended schedule of the available pharmaceutical formulations (Statement 9.3; Table S10). Additionally, the panel fully supported administering a booster dose of the MenACWY vaccine to preterm infants, following the minimum interval guidelines to ensure adequate protection (Statement 9.4; Table S10).

According to Canadian recommendations, stable premature infants should receive the conjugate meningococcal vaccine at the same chronological age and schedule as full-term infants [131].

The Four-Component meningococcal B (4CMenB) vaccine is regularly included in the immunization schedule of various countries [132]. However, when giving the primary immunization series to very preterm infants (≤ 28 weeks of gestation), especially those with a history of respiratory immaturity, the potential risk of apnea and the need for 48–72 h of respiratory monitoring should be taken into account. As the benefit of vaccination is high in this group of infants, vaccination should not be withheld or delayed [133].

A phase IV randomized study conducted in the UK showed that the 2 + 1 and 3 + 1 4CMenB vaccination schedules, followed by a booster, were immunogenic in 129 preterm infants. However, the 2 + 1 schedule exhibited a weaker response to strain NZ98/254 [134]. The panel asserts that meningococcal B (MenB) vaccines must be routinely administered to all preterm infants, with a two-dose primary series at 2 and 4 months of chronological age, followed by a booster dose at 12 months (Statement 9.5, 9.6, and 9.7; Table S10).

### Sector (10): Hib

Preterm and low birth weight infants are more likely to be hospitalized and to suffer from vaccine-preventable illnesses (VPDs), which can further increase morbidity and death. Therefore, there is a need to optimize protection from these diseases, which include pertussis, Hib, and invasive pneumococcal disease (IPD) [135].

Hib, which contains polyribosylribitol phosphate (PRP), is a leading cause of invasive infections, such as meningitis. The Hib vaccine has been widely used in many countries after its first introduction in 1988, leading to a significant reduction in the prevalence of Hib-related infectious diseases [136].

Many advantages are established regarding the combination vaccines, including a reduction in the number of injections and enhanced coverage duration [137].

A post-hoc analysis involving 111 preterm infants who were administered the Hexavalent (DTaP-IPV-HepB-Hib) vaccine in Phase II and III studies showed a low rate of adverse events. The vaccine not only exhibited an acceptable safety profile but also generated immune responses in premature infants that were satisfactory and similar to those observed in the overall study population [138].

Therefore, the panel recommended administering the Hib vaccine as part of Hexavalent formulations (DTaP-IPV-HB-Hib) rather than being administered alone for preterm infants, as long as the pertussis component is acellular (Statement 10.1; Table S11).

### Sector (11): Poliovirus vaccines

The panel endorsed that preterm infants receive three doses of the IPV vaccine at 2, 4, and 6 months of age to guarantee optimal protection (Statement 11.1; Table S12). Previous studies indicated that premature infants can generate immune responses comparable to those of term infants after receiving IPV for all three polio strains. When administered as part of a hexavalent vaccine, both preterm and term infants achieved 100% seroconversion for poliovirus serotypes I, II, and III. However, the geometric mean titers (GMTs) were significantly lower in preterm infants, with term-to-preterm GMT ratios of 1.8 (95% CI 1.28–2.61) for serotype I, 1.36 (95% CI 0.93–2.00) for serotype II, and 2.58 (95% CI 1.75–3.80) for serotype III [27]. Another study conducted in India indicated that IPV can be administered to preterm infants according to their chronological age, which can involve either a single intramuscular dose at 14 weeks or three IM doses at 6, 10, and 14 weeks, followed by a booster at 16 to 24 months [139].

In terms of Hexavalent vaccines, the panel unanimously recommended using the IPV vaccine within the Hexavalent (DTaP-IPV-HB-Hib) formulation over giving it alone for preterm infants (Statement 11.2; Table S12). Numerous studies acknowledged that Hexavalent (DTaP-IPV-HB-Hib) vaccines have an immunogenicity and safety profile similar to that of full-term infants. However, preterm infants could experience a rise in cardiorespiratory adverse events, including apnea, bradycardia, and desaturation, after vaccination [140–143].

The panel strongly recommended not to administer the OPV at the NICU before the time of discharge. This approach is aimed at mitigating the risk of live poliovirus circulation in vulnerable preterm infants (Statement 11.3; Table S12). The narrative review conducted by Sahoo et al. supports this statement, highlighting the importance of timing and setting for OPV administration to prevent the risk of enteral circulation and the transmission of the live poliovirus to other sick infants in the NICU [139].

More than 90% of the panel agreed that to reduce the risk of VAPP and circulating vaccine-derived poliovirus type 2 (cVDPV2), aligning with global strategies to phase out OPV and prioritize IPV in routine immunization programs, the current polio immunization schedule should be modified by dropping the zero dose (birth dose) and 2-month OPV and use IPV at 2 months, followed by IPV doses at 4, 6, and 18 months in addition to two OPV doses starting from the age of 4 months (Statement 11.4; Table S12).

### Sector (12): MMR vaccine

Antibodies transferred passively from an immunized mother usually offer adequate protection up to 6 months of age [144]. In the Netherlands, preterm infants are recommended to follow the same vaccination schedule of MMR vaccines as term infants, irrespective of their prematurity [145]. To ensure adequate protection against MMR, the panel suggested that preterm infants should be given two doses of the MMR vaccine at 12 and 18 months (Statement 12.1; Table S13).

However, administering the first dose at 6 months for high-risk preterm infants was not supported by the panel (Statement 12.2; Table S13). Vaccination with the MMR at 9 months of age or earlier has been shown to lead to insufficient seroconversion [146].

### Sector (13): Varicella vaccine

Administering the varicella vaccine at the recommended chronological age produces a sufficient antibody response to the vaccine antigens, even in infants born before 29 weeks of gestation [147]. In agreement with these guidelines, the panel supported administering the varicella vaccine at the suggested chronological age to ensure an adequate antibody response (Statement 13.1; Table S14). Studies showed that giving the chickenpox vaccine promptly can significantly reduce both the incidence and severity of varicella in preterm infants [148]. In 2006, the ACIP introduced a routine two-dose varicella vaccination schedule for children. They recommended giving the first dose at 12–15 months and the second dose between four and six years of age [149, 150].

### Sector (14): Post-vaccination adverse events

This sector highlights post-vaccination adverse events in preterm infants as well as the key concerns and challenges regarding safety and potential complications. Experts widely agree that minor side effects such as pain, redness, and swelling, as well as symptoms like low fever and irritability, occur equally in both preterm and full-term infants (Statement 14.1; Table S15). In a pilot study by Stevens et al. on 40 premature neonates, they found that preterm infants can express pain stimuli similarly to full-term neonates by bulging their brows, squeezing their eyes, furrowing their nasolabial, or even opening their lips, but factors altering this response need further research [151]. Shinefield et al. conducted a randomized trial on 37,868 infants receiving PCV; they revealed no significant changes in fever and local reactions after PCV vaccination between preterm and full-term infants. However, they noted increased redness and swelling among low-birth-weight infants. Moreover, there was more significant swelling in preterm infants after the third dose [152]. The panel clarified that further studies are warranted to better understand the reactogenicity in preterm infants for more reliable results.

75% of experts agreed that preterm infants may experience cardiorespiratory events like apnea and bradycardia after receiving the two-month vaccines. However, the incidence of these adverse events is lower with acellular pertussis-containing vaccines compared to whole-cell pertussis-containing vaccines (Statement 14.2; Table S15). A randomized clinical trial involving 223 preterm infants born at less 33 weeks’ gestational age found that 24% of vaccinated infants who received the two-month vaccinations experienced apnea within 48 h, compared to 10% in the unvaccinated group. However, the benefits of vaccination outweigh the risk of apnea [153]. In another randomized trial on 93 preterm infants (< 37 weeks) who received diphtheria-tetanus-aP vaccination and 98 infants as a control group who didn’t, the results indicated that preterm infants vaccinated at 2 months after birth did not have a higher incidence of prolonged apnea and bradycardia compared to those in the control group [154].

For those who were born extremely premature and required prolonged NICU stays (more than 2 months), the panel discussed whether they should be monitored carefully for possible cardiorespiratory events following vaccination. Some consultants recommended that all preterm infants should be observed in the NICU for 48 h after vaccination. In contrast, others argued about the applicability of this monitoring if the infant is stable, suggesting administering vaccinations 48 h before discharge rather than prolonging the hospital stay solely for observation. While extended stays in the NICU beyond 60 days are rare for preterm infants, the policies should aim for generalizability rather than exceptions. The potential risk of apnea and the need for respiratory monitoring for 48–72 h should be considered when administering the primary immunization series of MenB as well as DTaP-IPV-HB-Hib vaccine to very premature infants (born ≤ 28 weeks of gestation) and particularly for those with a previous history of respiratory immaturity. As the benefit of vaccination is high in this group of infants, vaccination should not be withheld or delayed [118, 133].

The panel recommends that stable preterm infants with bronchopulmonary dysplasia (BPD) who are stable at home should not delay vaccinations. For hospitalized preterm infants with BPD and cardiorespiratory events, the panel agreed that vaccination should be postponed as infants with BPD may face additional challenges, such as being on diuretics, steroids, or in an unstable condition (Statement 14.3.1 and 14.3.2; Table S15). They also recommended that hospitalized preterm infants should be continuously monitored for at least 48 h to 72 h following vaccination to identify and manage any cardiorespiratory adverse events (Statement 14.4; Table S15). Several studies recommend monitoring preterm infants for 48 to 72 h after immunization for the potential development of apnea or bradycardia [155–157].

A retrospective study by Montague et al. found that respiratory decompensation requiring clinical intervention after immunization was rare and did not significantly differ between preterm infants under 32 weeks with and without bronchopulmonary dysplasia. They recommended that immunizations for this at-risk group should proceed without delay [158].

Sudden infant death syndrome (SIDS) is defined as the sudden death of an infant under one year of age, which remains poorly understood despite thorough investigations [159]. 88% of the panel agreed that preterm infants are not associated with an increased risk of SIDS (Statement 14.5; Table S15). In a large case-control study, Vennemann et al. found no increased risk of SIDS in vaccinated infants. In fact, the study highlighted the protective role of vaccination. They explained that vaccination shields infants against unrecognized whooping cough, which is a well-established cause of both sudden and unexpected death in this age group; moreover, cross-immunization with other bacteria or viruses could further enhance the protection of infants [160]. Jonville-Béra et al. found that vaccination with DTPP ± Hib in preterm infants wasn’t a risk factor for early substance use disorder (SUD) or SIDS [161]. They identified several significant risk factors for early SIDS, including low birth weight, sleeping in a prone position, lack of breastfeeding, multiple births, and parental smoking.

A range of local and systemic reactions could occur after vaccination. Administering analgesics or antipyretics as a preventive measure can reduce adverse reactions after vaccination [162]. However, the panel advises against routinely using these medications before giving vaccinations to extremely premature infants (Statement 14.6; Table S15). A systematic review shows that giving prophylactic antipyretics can relieve local and systemic symptoms after primary vaccinations; however, it may also reduce antibody responses to various vaccine antigens without affecting the immunogenicity [163].

Research indicates that preventive use of acetaminophen and ibuprofen can lessen negative reactions in young infants receiving the wP vaccine. However, with the transition from DTwP to DTaP, no advantages have been identified for this vaccine in children aged 4–6 or with other vaccines [164].

### Sector (15): Indirect protection and family immunization

Family members and caregivers of infants must keep their vaccinations updated to ensure a protective immunity barrier around infants, safeguarding their health from severe infections [25]. The panel strongly recommended that parents, siblings, caregivers, and healthcare providers of preterm infants should be routinely immunized to reduce the risk of transmission. In certain regions, the unavailability of adult vaccination remains a significant challenge. Tdap-containing vaccines are the preferred option, and ensuring their accessibility is critically important (Statement 15.1; Table S16).

Maternal immunization is vital for protecting pregnant women and young infants from serious infectious diseases [165]. The panel strongly recommended that pregnant women receive Tdap or Tdap-IPV, along with influenza vaccines, during prenatal care to protect both themselves and their infants (Statement 15.2; Table S16). This is also recommended in the USA for all pregnant women [165]. The panel also strongly supported educating families about vaccination during a preterm infants’s hospital stay, as it plays a crucial role in improving adherence to the infant’s immunization schedule (Statement 15.3; Table S16). Gagneur et al. emphasized that educating families on the importance of timely vaccinations enhances adherence to immunization schedules [166]. The summary of recommendations is presented in Table 1.

## Conclusion

Preterm birth has become increasingly common over the past several decades. The underdeveloped immune systems place preterm infants at an increased risk of infections, often vaccine-preventable, compared to full-term infants. This consensus provides structured, evidence-based recommendations incorporating local perspectives for optimizing vaccination strategies in preterm infants in Egypt.

The findings highlight the importance of timely and complete vaccination to reduce infection-related morbidity and mortality in preterm infants. While consensus was achieved on most statements, some areas remained unresolved, highlighting the need for further research and continuous updates to vaccination guidelines. The main limitation of this consensus is the absence of cost-effectiveness studies on the immunization measures among preterm infants in Egypt. Future efforts should focus on long-term vaccine effectiveness, safety, and immunogenicity in preterm infants, especially in Egypt.

## Supplementary Information

Below is the link to the electronic supplementary material.

Supplementary Material 1

## Data Availability

Data sharing is not applicable to this article as all data generated and analysed during the current study are included in this article.

## Abbreviations

4CMenB: Four-component meningococcal B
AAP: American academy of pediatrics
ACIP: Advisory committee on immunization practices
aP: Acellular pertussis
BCG: Bacillus calmette-guérin
BPD: Bronchopulmonary dysplasia
DTaP: Diphtheria, tetanus, and acellular pertussis
EPI: Expanded programme on immunization
FDA: Food and drug administration
HBIG: Hepatitis B immunoglobulin
HBsAg: Hepatitis B surface antigen
HBV: Hepatitis B virus
Hib: Haemophilus influenzae type B
HIV: Human immunodeficiency virus
IMD: Invasive meningococcal disease
IPV: Inactivated poliovirus vaccine
LMICs: Low- and middle-income countries
mAbs: Monoclonal antibodies
MENA: Middle East and North Africa
MenACWY: Meningococcal conjugate vaccine
MenB: Meningococcal B
MMR: Measles-mumps-rubella
MNT: Maternal and neonatal tetanus
MOHP: Ministry of health and population
NICU: Neonatal intensive care unit
LAIV: live-attenuated influenza vaccine
OPV: Oral polio vaccine
PCV: Pneumococcal conjugate vaccine
RSV: Respiratory syncytial virus
SIDS: Sudden infant death syndrome
UNICEF: United Nations Children’s Fund
VAPP: Vaccine-associated paralytic poliomyelitis
VLBW: Very-low-birth-weight
WHO: World Health Organization
wP: Whole-cell pertussis
