# Supplementary material for: Standardized vaccination practices for preterm infants: Egyptian experts’ consensus
Source: BMC Infect Dis. 2025 Dec 19;26:130. doi: 10.1186/s12879-025-12243-0 (PMC12831249; doi:10.1186/s12879-025-12243-0)
Supplement: Supplementary file 1 — Supplementary Material 1 [file 12879_2025_12243_MOESM1_ESM.docx]

**Supplementary Material 1**

**Table S1. Consensus Statements on Vaccination Challenges and Considerations for Preterm Infants**

| **Sector 1: Vaccination Challenges and Considerations for Preterm Infants** | | |
| --- | --- | --- |
| **Statement** | **Agreement percentage** | **Agreement level** |
| - 1. Chronological age is preferred over corrected age for determining the timing of vaccinations in preterm infants. | 100% | I |
| - 1. Preterm infants should follow the same vaccination schedule as term infants, regardless of corrected age. | 100% | I |
| - 1. Vaccination should be postponed if the preterm infant presents with unstable hemodynamic conditions, sepsis, or infectious or neuro-metabolic disorders. | 100% | I |
| - 1. Intramuscular (IM) vaccines can be administered to preterm infants at their chronological age, provided their weight ≥ 2000 grams, except for Hepatitis B Vaccine. | 70% | II |
| - 1. The vastus lateralis muscle is preferred for intramuscular injections in infants due to its larger muscle mass. | 94% | I |
| - 1. Vaccine site preference shouldn’t differ for preterm infants with very low birth weight (<1500 g) compared to those closer to term weight. | 89% | II |
| - 1. Shorter needles (<16 mm) should be used for intramuscular vaccinations in preterm infants to accommodate reduced muscle mass | 94% | I |
| - 1. Vaccination should be avoided in areas of skin lesions, bruising, or scarring in preterm infants. | 94% | I |
| 1.9.1. Live virus vaccines, such as rotavirus vaccines, should not be used in hospital environments where there are immunocompromised individuals. | 83 % | II |
| 1.9.2. Live virus vaccines, such as OPV, should not be used in hospital environments where there are immunocompromised individuals. | 100% | I |

**I**: ≥90% agreement; **II**: ≥70% but <90% agreement.

**Table S2. Consensus Statements on BCG Vaccine**

| **Sector 2: BCG Vaccine** | | |
| --- | --- | --- |
| **Statement** | **Agreement percentage** | **Agreement level** |
| - 1. BCG vaccination should be delayed in preterm infants born before 34 weeks of gestation until they reach 34 weeks of gestational age. | 94% | I |
| - 1. BCG vaccination should be postponed in preterm infants weighing less than 2000 grams. | 100% | I |
| - 1. BCG vaccination guidelines should be modified for preterm infants born to mothers who used immunosuppressants. | 82.4% | II |
| - 1. Given the reported immunogenicity benefits of BCG in preterm infants, early administration is not justified to enhance resistance to respiratory infections and neonatal sepsis. | 72% | II |

**I**: ≥90% agreement; **II**: ≥70% but <90% agreement.

**Table S3. Consensus Statements on Hepatitis B Vaccine**

| **Sector 3: Hepatitis B Vaccine** | | |
| --- | --- | --- |
| **Statement** | **Agreement percentage** | **Agreement level** |
| - 1. Preterm infants with HBsAg-unknown mothers should be treated as HBsAg positive, receiving HBIG and the monovalent hepatitis B vaccine within 12 hours of birth to be followed by another three doses. | 94% | I |
| - 1. Preterm infants with HBsAg-unknown mothers should be managed as HBsAg-positive, receiving HBIG and the hepatitis B vaccine within 12 hours of birth. | 94% | I |
| - 1. Infants weighing less than 2000 g should follow an adjusted hepatitis B vaccine schedule based on their birth weight and clinical stability. | 94% | I |
| - 1. Serology testing for HBsAg and anti-HBs should be conducted at 9-12 months of age for preterm infants with HBsAg-positive or unknown mothers. | 94% | I |
| - 1. HepB in combination vaccines and separately administered HepB vaccines are safe in preterm infants (booster dose + combo) | 89% | II |

**I**: ≥90% agreement; **II**: ≥70% but <90% agreement.

**Table S4. Consensus Statements on Respiratory Syncytial Virus Disease**

| **Sector 4: Respiratory Syncytial Virus Disease** | | |
| --- | --- | --- |
| **Statement** | **Agreement percentage** | **Agreement level** |
| - 1. RSV-specific mAb antibody should be administered to preterm infants born during or before the RSV season for direct protection from RSV disease. | 100% | I |
| - 1. Extended half-life mAbs (Nirsevimab) is preferred over short-acting mAb (palivizumab) for protection against RSV in preterm infants, when both options are available. | 94% | I |

**I**: ≥90% agreement; **II**: ≥70% but <90% agreement.

**Table S5. Consensus Statements on Pneumococcal Vaccines**

| **Sector 5: Pneumococcal Vaccines** | | |
| --- | --- | --- |
| **Statement** | **Agreement percentage** | **Agreement level** |
| 5.1. Preterm infants should receive 4 doses of Pneumococcal Conjugate Vaccine (PCV) according to the recommended schedule (2, 4, 6, and 12-15 months). | 94% | I |
| 5.2. Preterm infants should receive additional pneumococcal vaccine doses beyond the age of 5 under certain conditions (e.g., immunocompromised status, chronic medical conditions. | 89% | II |
| 5.3. Preterm infants should receive 2 doses of 23vPPV at 4 and 9 years, as per the recommended pneumococcal vaccination schedule. | 94% | I |

**I**: ≥90% agreement; **II**: ≥70% but <90% agreement.

**Table S6. Consensus Statements on Rotavirus Vaccine**

| **Sector 6: Rotavirus Vaccine** | | |
| --- | --- | --- |
| **Statement** | **Agreement percentage** | **Agreement level** |
| - 1. Oral live attenuated rotavirus vaccine is contraindicated in preterm infants with suspected immunodeficiency or whose mothers used biologicals during pregnancy. | 94% | I |
| - 1. The first dose of the rotavirus vaccine should be administered to healthy preterm infants between 6 and 15 weeks of chronological age. | 88% | II |
| - 1. The oral live attenuated rotavirus vaccine is contraindicated for hospitalized age-eligible preterm infants in a hospital environment. | 72% | II |
| - 1. Preterm infants, discharged in time of RV vaccination, should follow the same immunization schedule as full-term infants. | 100% | I |

**I**: ≥90% agreement; **II**: ≥70% but <90% agreement.

**Table S7. Consensus Statements on Seasonal Influenza Vaccine**

| **Sector 7: Seasonal Influenza Vaccine** | | |
| --- | --- | --- |
| **Statement** | **Agreement percentage** | **Agreement level** |
| - 1. Preterm infants equal to or above the age of 6 months should receive two doses of the influenza vaccine, one month apart, in the first year, followed by one dose annually in subsequent years. | 94% | I |

**I**: ≥90% agreement; **II**: ≥70% but <90% agreement.

**Table S8. Consensus Statements on DTP-containing vaccine**

| **Sector 8: DTP-containing vaccine** | | |
| --- | --- | --- |
| **Statement** | **Agreement percentage** | **Agreement level** |
| - 1. Based on the current evidence, the acellular pertussis vaccine offers a better safety profile for preterm infants compared to the whole-cell pertussis vaccine. | 100% | I |
| - 1. Acellular pertussis vaccines should be preferred over whole-cell pertussis vaccines for preterm infants regardless of their weight and chronological age | 100% | I |
| - 1. Higher-valency vaccines, such as Hexavalent (DTaP-IPV-HB-Hib) formulations, are preferred over lower-valency or separate vaccines to minimize the number of injections required for preterm infants. | 100% | I |
| - 1. Preterm infants should receive three doses of diphtheria, tetanus, acellular pertussis, IPV, Hib, and HepB vaccine at 2, 4, and 6 months of age according to chronological age. | 100% | I |
| - 1. Preterm infants should receive a booster dose of diphtheria, tetanus, acellular pertussis, IPV, Hib, and HepB vaccine at 12-18 months of age. | 100% | I |

**I**: ≥90% agreement; **II**: ≥70% but <90% agreement.

**Table S9. Consensus Statements on Meningococcal Vaccines**

| **Sector 9: Meningococcal Vaccines** | | |
| --- | --- | --- |
| **Statement** | **Agreement percentage** | **Agreement level** |
| - 1. No polysaccharide vaccine can be given below the age of 2 years. Any infant, regardless of gestational age, should receive conjugate meningococcal vaccine as per the insert leaflet of each product | 100% | I |
| - 1. Conjugate meningococcal vaccines (e.g., MenACWY) should be routinely administered to preterm infants. | 94% | I |
| - 1. Preterm infants should receive the primary series of the meningococcal conjugate vaccine during the first year of life as early as the recommended schedule of the available pharmaceutical formulations. | 94% | I |
| - 1. Preterm infants should receive a booster dose of the meningococcal conjugate vaccine (MenACWY) with respect to the minimal interval. | 94% | I |
| - 1. Meningococcal B (MenB) vaccines are recommended to be routinely administered to preterm infants | 72% | II |
| - 1. Preterm infants should receive a two-dose primary series of meningococcal B vaccines at 2 and 4 months of chronological age. | 72% | II |
| - 1. Preterm infants should receive a booster dose of meningococcal B vaccines at 12 months of chronological age. | 83% | II |

**I**: ≥90% agreement; **II**: ≥70% but <90% agreement.

**Table S10. Consensus Statements on H. Influenza Type B Vaccine**

| **Sector 10: H. Influenza type B vaccine** | | |
| --- | --- | --- |
| **Statement** | **Agreement percentage** | **Agreement level** |
| 10.1. Using the Hib vaccine as part of Hexavalent (DTaP-IPV-HB-Hib) formulations is preferred over administering the Hib vaccine alone for preterm infants, provided that the pertussis component is acellular. | 94% | I |

**I**: ≥90% agreement; **II**: ≥70% but <90% agreement.

**Table S11. Consensus Statements on Poliovirus Vaccines**

| **Sector 11: Poliovirus Vaccines** | | |
| --- | --- | --- |
| **Statement** | **Agreement percentage** | **Agreement level** |
| - 1. Preterm infants should receive three primary doses of IPV vaccine at 2, 4, and 6 months of age. (booster dose). | 100% | I |
| - 1. Using the IPV vaccine as part of Hexavalent (DTaP-IPV-HB-Hib) formulations is preferred over administering the IPV vaccine alone for preterm infants. | 100% | I |
| - 1. Administering OPV just at discharge is preferable to administering it in the NICU to provide herd immunity & gut priming while avoiding the enteral circulation of live poliovirus among premature, sick infants. | 82% | II |
| - 1. To reduce the risk of vaccine-associated paralytic polio (VAPP) and circulating vaccine-derived poliovirus type 2 (cVDPV2), aligning with global strategies to phase out OPV and prioritize IPV in routine immunization programs, The current polio immunization schedule should be modified by dropping the zero dose (birth dose) and 2-month oral polio vaccine (OPV) and use inactivated polio vaccine (IPV) at 2 months, followed by IPV doses at 4, 6, and 18 months in addition to two OPV doses starting from the age of 4 months. | 94% | I |

**I**: ≥90% agreement; **II**: ≥70% but <90% agreement.

**Table S12. Consensus Statements on MMR Vaccine**

| **Sector 12: Measles Mumps Rubella (MMR) Vaccine** | | |
| --- | --- | --- |
| **Statement** | **Agreement percentage** | **Agreement level** |
| - 1. Preterm infants should receive two doses of MMR vaccine at 12 and 18 months of age. | 100% | I |
| - 1. The first dose of the MMR vaccine should not be administered as early as 6 months instead of 12 months in high-risk preterm infants to account for the early loss of maternal antibodies. | 100% | I |

**I**: ≥90% agreement; **II**: ≥70% but <90% agreement.

**Table S13. Consensus Statements on Varicella Vaccine**

| **Sector 13: Varicella Vaccine** | | |
| --- | --- | --- |
| **Statement** | **Agreement percentage** | **Agreement level** |
| - 1. Administering varicella vaccine at the recommended chronological age results in an adequate antibody response. | 100% | I |

**I**: ≥90% agreement; **II**: ≥70% but <90% agreement.

**Table S14. Consensus Statements on Post-Vaccination Adverse Events**

| **Sector 14: Post-Vaccination Adverse Events** | | |
| --- | --- | --- |
| **Statement** | **Agreement percentage** | **Agreement level** |
| 14.1. Low-grade side effects like pain, redness, swelling, or symptoms like low fever and irritability occur equally in preterm and full-term infants. | 94% | I |
| 14.2. Premature infants may experience cardiorespiratory events like apnea and bradycardia after receiving the two-month vaccines. However, the incidence of these adverse events is lower with acellular pertussis-containing vaccines compared to whole-cell pertussis-containing vaccines. | 75% | II |
| 14.3.1. Vaccinations should not be postponed in preterm infants who are stable at home with bronchopulmonary dysplasia and cardiorespiratory events. | 78% | II |
| 14.3.2. Vaccinations should be postponed in preterm infants who are hospitalized with bronchopulmonary dysplasia and cardiorespiratory events. | 100% | I |
| 14.4. It is recommended to monitor hospitalized preterm infants for apnea or bradycardia for up to 48-72 hours after vaccination. | 94% | I |
| 14.5. Vaccination in preterm infants is not associated with an increased risk of SIDS. | 88% | II |
| 14.6. Prophylactic analgesics or antipyretics should not be administered before vaccinations in extremely premature infants to reduce the risk of pain and fever. | 72% | II |

**I**: ≥90% agreement; **II**: ≥70% but <90% agreement.

**Table S15. Consensus Statements on Indirect Protection and Family Immunization**

| **Sector 15: Indirect Protection and Family Immunization** | | |
| --- | --- | --- |
| **Statement** | **Agreement percentage** | **Agreement level** |
| - 1. Parents, siblings, caregivers, & HCPs interacting with preterm infants should be routinely immunized to reduce the risk of transmission. In certain regions, the unavailability of adult vaccination remains a significant challenge. Tdap-containing vaccines are the preferred option, and ensuring their accessibility is critically important. | 93% | I |
| - 1. Pregnant women should receive Tdap or Tdap-IPV, along with the influenza vaccine, during prenatal care to protect both themselves and their infants. | 100% | I |
| - 1. Providing vaccination education to families during a preterm infant’s hospital stay increases adherence to the infant's immunization schedule. | 94% | I |

**I**: ≥90% agreement; **II**: ≥70% but <90% agreement.
